# High Precision Location Estimation in Mountainous Areas Using GPS

**DOI:** 10.3390/s22031149

**Published:** 2022-02-02

**Authors:** Yugo Kunisada, Chinthaka Premachandra

**Affiliations:** Department of Electrical Engineering and Computer Science, Graduate School of Engineering and Science, Shibaura Institute of Technology, Tokyo 1358548, Japan; ma20035@shibaura-it.ac.jp

**Keywords:** GPS, GNSS, data science, location estimation

## Abstract

Outdoor recreation has become popular in recent years, against the backdrop of the new coronavirus epidemic that started in 2020. Mountaineering, in particular, has become a popular pastime for many people as an easy way to experience nature. However, the number of mountaineering accidents is increasing, owing to the inadequate knowledge and equipment for beginners. In particular, the lack of map-reading skills and experience often leads to the selection of wrong trails. The smartphones used for precise location information obtain correction information from radio waves from a base station, and the accuracy of using only the GPS in mountainous areas without radio waves is questionable. In general, the GPS position correction methods in the literature for such situations include complex processing of the GPS radio waves. Some of these methods have been proposed with complex hardware and are difficult to implement with portable hardware. In this study, we develop and demonstrate a method for obtaining accurate location information using GPS without the error correction of radio waves, even in mountainous areas. The multipath is the reason for most of the GPS errors in the mountains. In the mountains, depending on the locations, the correct GPS location can also be received. In the proposed method, the correct GPS data are used to detect the incorrect GPS locations. We present an experimental method for estimating the interrelationship between the GPS longitude and latitude data. Additionally, we demonstrate the effectiveness of our method by showing that the experimental mountain location data presented in this paper are more accurate than the GPS data alone.

## 1. Introduction

**Figure 1:**
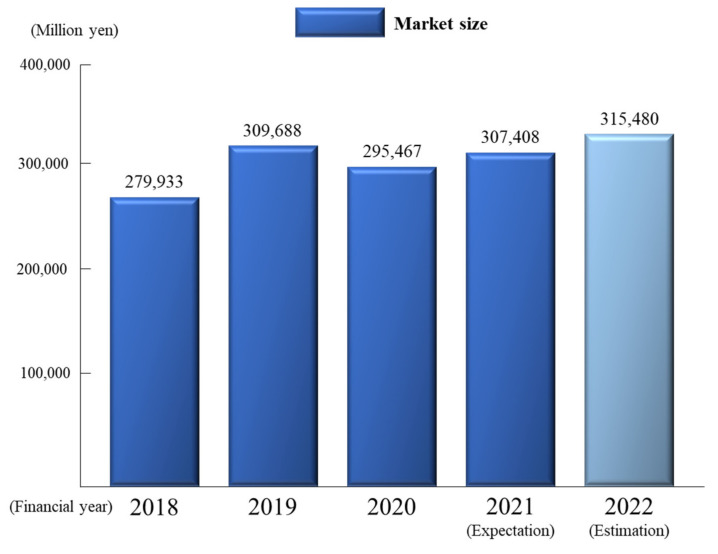
Trends and forecasts of the domestic outdoor equipment, facilities, and rental market [2].

In recent years, owing to the global outbreak of a new coronavirus and to aid the prevention of the spread of infection, entertainment activities such as visiting restaurants, theme parks, and traveling overseas have been greatly restricted, and their vitality is lost. Meanwhile, outdoor activities, which involve less human interaction and a lower risk of infection, have become more popular among both young and old people, made accessible by the outdoor activity boom, which occurred prior to the coronavirus outbreak, and technological improvements in equipment [1] (Figure 1: Trends and forecasts of domestic outdoor equipment, facilities, and rental markets [2]). Mountaineering, in particular, has gained popularity as a leisure activity that can be enjoyed easily by anyone because of the ease of access to well-maintained mountain trails, the improvement in the quality of mountain lodge accommodation, and the lightening of mountain equipment.

While it has become easier for beginners to enjoy mountain climbing, there has been a continuous increase in the number of mishaps owing to inappropriate clothing, equipment, or lack of planning and preparation [3]. According to the “Summary of mountain mishaps in 2020,” published by the Life Safety Planning Division of the Life Safety Bureau of the National Police Agency (Japan), the number of mishaps was 2294, which, although decreased from the previous year, was higher than the number of mishaps in the past 10 years.

One of the reasons for this high incidence of distress in mountainous areas is a lack of map-reading ability, especially in the case of novice climbers. In recent years, the widespread use of smartphones has made it easy to obtain accurate information about one’s location using map applications and GPS functions on smartphones. Therefore, many mountaineers now use their smartphones to navigate when climbing mountains, eliminating the need to read maps. However, the accuracy of GPS on a smartphone is unclear [4], because it is based on satellite position corrections sent from the nearest mobile phone base station [5], and these corrections are used to calculate the exact position of the smartphone. Map applications are designed to be used online and cannot be used offline. In Japan, where the terrain is predominantly mountainous, there are many mountain areas where mobile phone signals are unreachable, making it difficult to obtain precise location information using only a smartphone.

**Figure 2:**
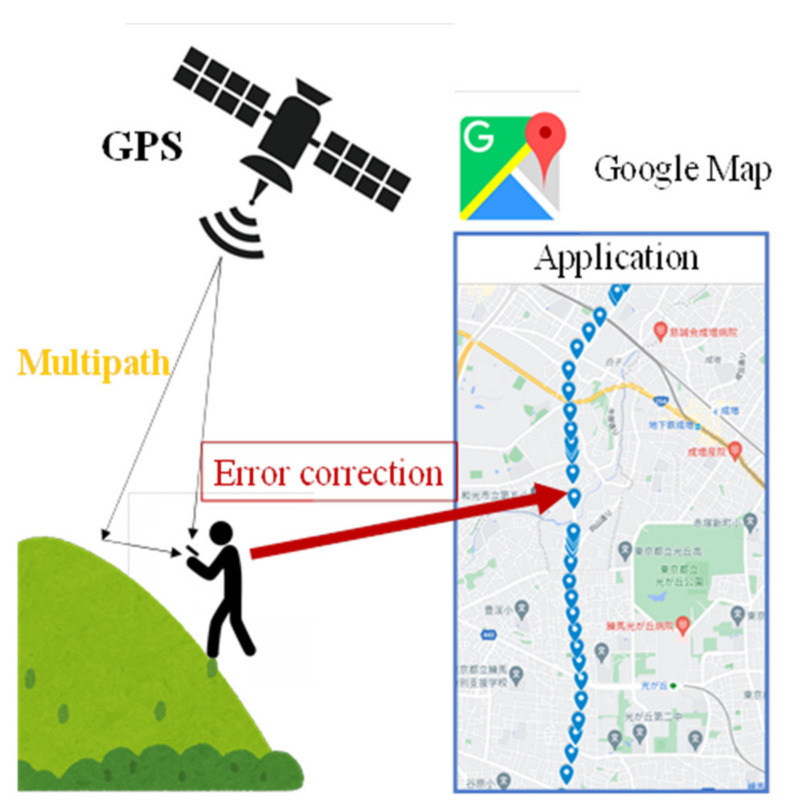
The outline of this study.

Therefore, in this study, we tackle detecting accurate location estimation in mountainous areas. The multipath is the reason for most GPS errors in the mountains. In the mountains, depending on the location, the correct GPS location can also be received. In the proposed method, these correct GPS data are used to detect the incorrect GPS locations. The outline of this study is presented in Figure 2. In mountainous areas, especially in the valleys between mountains, it is difficult to interpret GPS signals accurately because of multipath propagation in the mountains and poor visibility [3]. Under these circumstances, GPS position information is inaccurate with several errors. The method involves accumulating GPS position data, including errors in a database at all times, and calculating the best value from the correct position data available.

This method is novel and based on statistical inference of the acquired GPS data, rather than developing and adapting an effective algorithm to the signal [6,7,8,9,10], as in the case of the a posteriori multipath estimator technique (APME) [11] developed by Septentrio. There are also several methods of position estimation based on data, but these are for waveforms data and are different from this proposed method [12,13]. Similarly, there is a method of location estimation using machine learning from a database, but this method is also performed on waveforms data and is different from our method [14,15].

In this study, we have (a) programmed and built a GPS logger system; (b) checked the accuracy of our GPS system in use in a mountainous area; and (c) estimated the positional information based on the results and examined whether the results were reasonable.

## 2. System Overview

**Figure 3:**
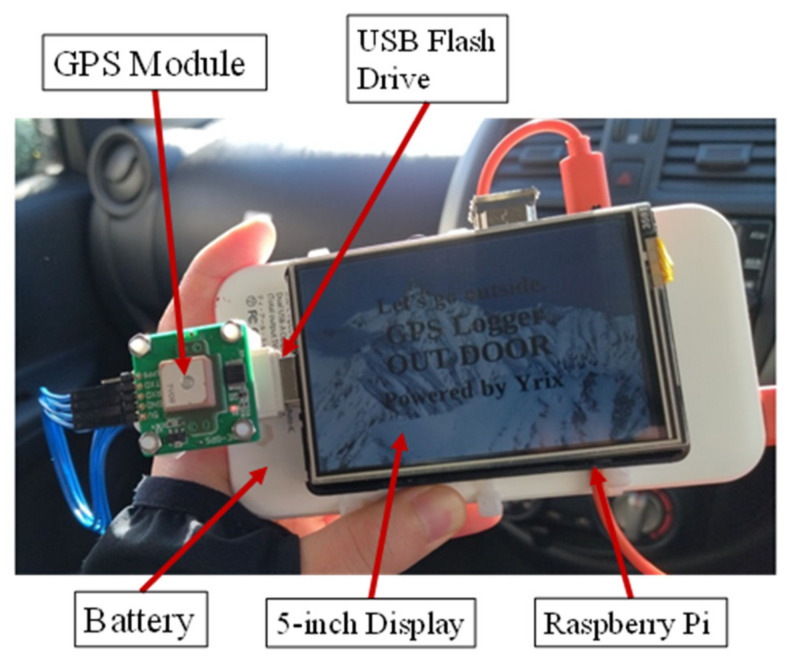
GPS logger that we developed and used in this study.

In this study, we first programmed and fabricated a GPS logger system. Figure 3 shows the GPS logger that we developed and used in this study.

**Figure 4:**
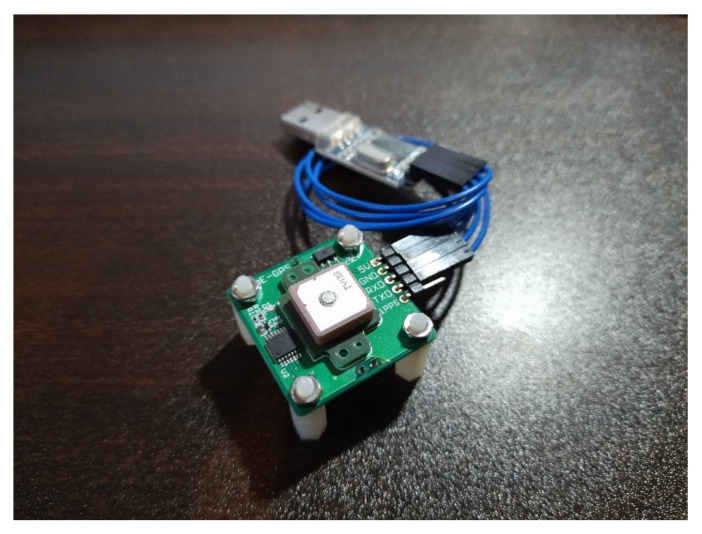
A GPS receiver kit sold by Akizuki Denshi Tsusho [5].

The GPS logger used in this study was a GPS receiver kit (Akizuki Denshi Tsusho Co., Ltd., Tokyo, Japan) [16] (Figure 4), which uses GYSFDMAXB (TAIYO YUDEN CO., LTD., Tokyo, Japan) [17] as the GPS module. The receiver is compatible with three Japanese Quasi-Zenith Satellite Systems (QZSS): “MICHIBIKI” (satellite numbers 193 (MICHIBIKI first), 194 (MICHIBIKI second), and 195 (MICHIBIKI fourth)). Reception accuracy was improved for use in Japan. The receiving frequency is 1575.42 (MHz, L1, C/A code) and the modulation method is BPSK. The National Marine Electronics Association 0183 (NMEA) sentences used in this study were the GPGGA. Therefore, latitude, longitude, time, and altitude were obtained by serial communication using this module.

A Raspberry Pi 3 Model B+ (Raspberry Pi Foundation, Cambridge, England, UK) was used as the single-board computer to perform the processing. Table 1 shows the specifications of the Raspberry Pi 3 Model B+; the latest version of the Raspberry Pi is the 4 Model B. Although the specifications of the Raspberry Pi 4 Model B have improved with the 3 Model B+, the power consumption has increased significantly. In this study, we used 3 Model B+ in consideration of mobility. The capacity of the mobile battery used was 20,000 (mAh), and the continuous user time was approximately 6 h.

**Figure 5:**
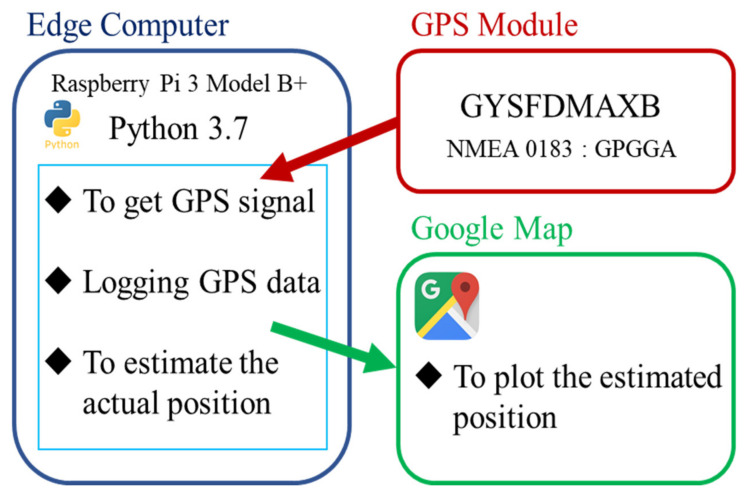
A schematic illustration of our system.

The programming language used to build the system was Python 3.7, chosen because of its rich library, ease of access to network communication, and its potential for future development. In this study, we used Google Maps to display a point then compare the behavioral records and the estimated locations. The system of this study observes the longitude and latitude every 10 s and stores this data in the database. The name of the data is expressed in time, and it is possible to display up to 6 h and 35 min of the data at one time on Google Maps. Figure 5 shows a schematic of the proposed system.

## 3. Method

### 3.1. GPS Data Analysis

In this study, we first examined and discussed the results, accuracy, and sources of error when using only GPS location information and actual measurements with the device. The plotted location information was updated and stored every 10 s, and all information was acquired while walking. In the system, the signals sent from the GPS module to the edge computer are truncated to two decimal places, so there is always an error compared to the actual received signals. Therefore, all errors within a radius of 5 m were assumed to be negligible.

**Figure 6:**
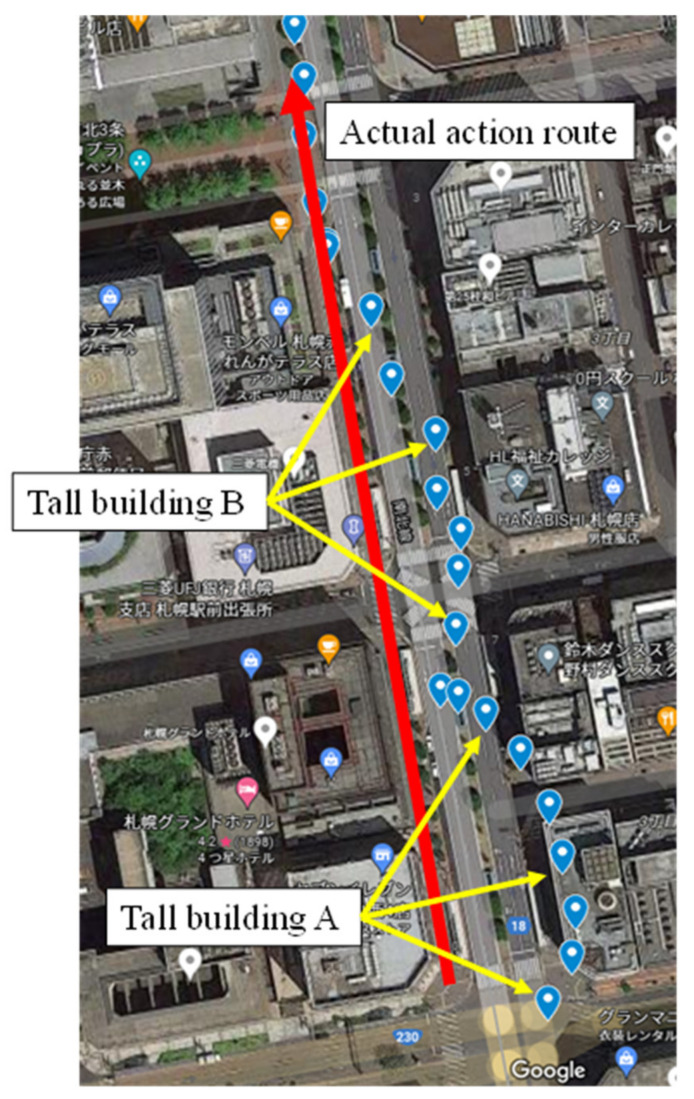
GPS location and its error in urban area (Sapporo, Hokkaido, Japan).

Data were collected using the proposed research device, and the experiment was conducted in a group of buildings in an urban area. The data showed that, overall, location information was recorded accurately and that the current use of the device is sufficient to check the past activity records. However, multipath errors are likely to occur between tall buildings, with errors of up to 25 m or more, or in areas with poor visibility (Figure 6). This is because the accuracy of GPS position information is guaranteed by four satellite signals; therefore, it is expected to reflect large positional errors in locations where such reflected waves occur, or in environments where one or two satellite signals cannot be received [18,19].

**Figure 7:**
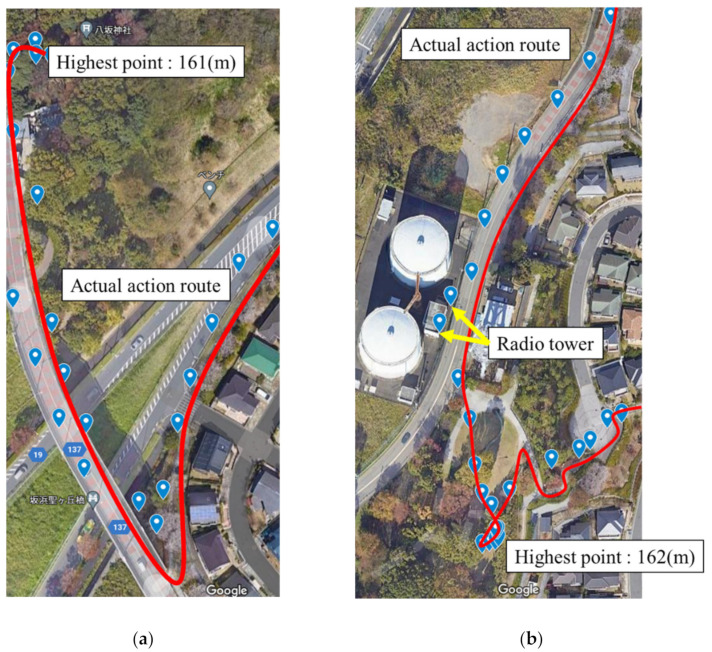
GPS location and its error in each area. (**a**) GPS location in a relatively high-altitude area with good visibility (Yasaka Shrine, Tama City, Tokyo: 161 m above sea level). (**b**) GPS position information and its error near a wireless base station tower (Inagi, Tokyo, Japan, at an elevation of 162 m).

Next, we recorded a route at a relatively high altitude with good visibility. The distance traveled was approximately 3.5 km, and the altitude was measured as between 40 m and 166 m. Here, we obtained location information with a very high accuracy (Figure 7a). We were able to receive position information with a maximum error of 10 m for all data. However, the accuracy of the location information deteriorated significantly near a mobile phone base station and a disaster prevention radio base station, which were located near the highest elevation point, owing to interference and an increase in noise floor level (Figure 7b). Table 2 illustrates the numerical data of the error with the respective latitude/longitude information.

Based on these results, it is concluded that the location error in mountainous areas is caused by a multipath issue in the valleys between the mountains, interference near disaster prevention radios, mobile phone base stations, or broadcasting towers located near mountain peaks and at relatively high altitudes; in addition, there is a need to estimate the location against the GPS signal interference.

### 3.2. Estimate Position Processing

As a prerequisite for this study, we assume that the GPS location information is recorded at one point every 10 s and that the user is a pedestrian. Under this assumption, the difference in the distance traveled in 10 s is not as large as it would be for vehicles such as cars or trains. Errors due to the multipath of the GPS signal and large deviations of the receiving point owing to interference, which are both real problems, cause a difference in distance that is not possible in a 10-s walk. Therefore, by considering the difference between the previous and next acquired position data, we can estimate the correct location. This can be expressed as Equation (1):

(1) $$\begin{array}{c} Longitude:x_{n}=\frac{\left(x_{n+1}-x_{n-1}\right)}{n}+x_{n+1}\\ Latitude:y_{n}=\frac{\left(y_{n+1}-y_{n-1}\right)}{n}+y_{n+1} \end{array}$$

However, it is difficult to obtain an accurate value with a simple calculation such as Equation (1) because the output depends mainly on the previous and subsequent data. In fact, if the values of the previous and subsequent data contain a large error, incorrect position information will be obtained. In addition, in mountainous areas, especially near the floors of valleys prior to climbing up a ridge, trails are often complicated, and the above formula would be not be accurate in coping with the large changes in the direction of travel in 10 s.

**Figure 8:**
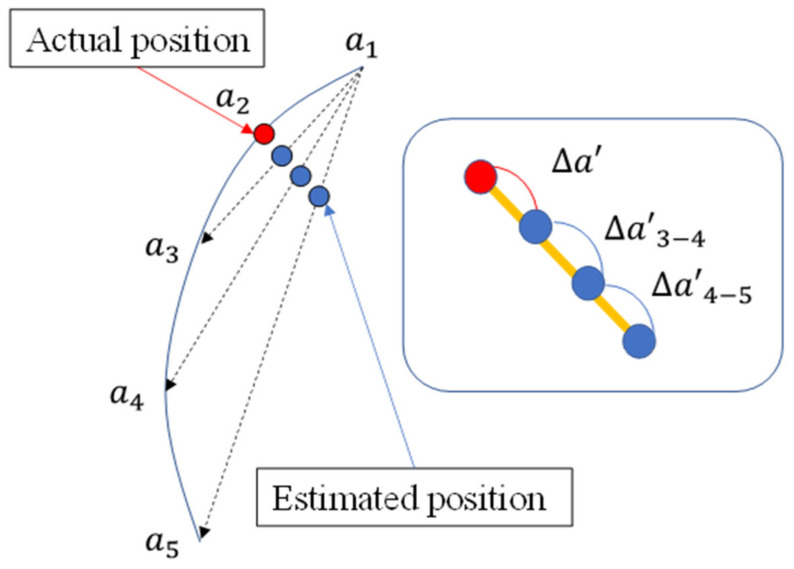
Overview of the method used to calculate the position estimates in this study.

Therefore, in this study, we propose a new method for estimating the past location based on previous information, using the location information of up to five points. Let the point to be predicted be $a_{2}$. The estimated position, $a_{2}^{n’}$, is calculated by the difference of each point $a_{1}$ to $a_{5}$ using Equation (1). Then, the distance between each of the estimated positions $a_{2}^{n’}$ is calculated and the average is deduced. This value is then added to $a_{2}^{1’}$ to obtain the predicted position $a_{2}^{’}$. This is illustrated in Equation (2) and Figure 8.

(2) $$\begin{array}{c} a_{2}^{’}=a_{2}^{1’}+∆a^{’}\\ ∆a^{\prime }=\frac{\left(∆a{’}_{3-4}+∆{a^{\prime }}_{4-5}\right)}{2} \end{array}$$

**Figure 9:**
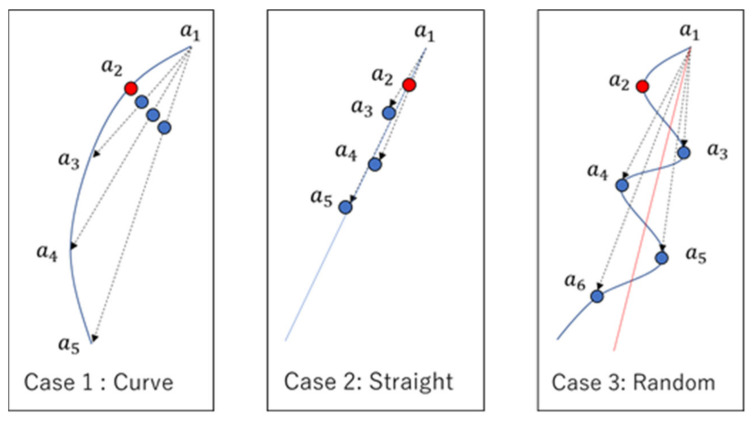
Three patterns of the estimated value in this study using Equation (2).

When Equation (2) is used, the estimated value can be classified into three patterns (Figure 9). In Case 1 (a curve) and Case 2 (a straight line), the difference can be calculated appropriately, so the method is effective. However, it is difficult to estimate the position of randomly scattered GPS locations, as in Case 3. However, Case 3 can be disregarded because it is unlikely that the user would be walking along a mountain path that meanders every 10 s and that the GPS would receive the information properly.

Equation (2) is not valid when the movement is zero, that is, when the user stops, for example, to rest. However, this process is not necessary during long rests or when movement has stopped because the GPS signal is received constantly and is more accurate. The estimation process at each point starts after the acquisition of sufficient data, that is, after 50 s. There are five reference points to be handled, because three points are insufficient for the calculation, and because the distance travelled varies greatly in situations where six or more points are required.

## 4. Experimental Evaluation

### 4.1. Experimental Enviroment

In this study, we used this experimental device to go mountain climbing. In the experiment, we recorded the location, time, and elevation of the route that we actually traveled based on a topographic map from the Geospatial Information Authority of Japan, and, at the same time, we acquired location information using GPS with only the experimental device and with the location information estimation system. Finally, we compared the data to examine the usefulness of this research.

The experimental site is located at Mt. Kentoku-yama (Japan) in the central part of the former Mitomi village, in the northern part of Yamanashi City, Yamanashi Prefecture. The altitude was 2031 m. It is surrounded by 2000-m peaks, such as Mt. Kasamori (2072 m) and Mt. Kurogane (2232 m), which makes it more difficult to receive GPS signals in comparison to higher mountains. The total height difference between the trailhead and the summit of Mt. Kentoku-yama was 1366 m, the distance was 10.8 km, and the total recording time was 6 h.

### 4.2. Experimental Results

**Figure 10:**
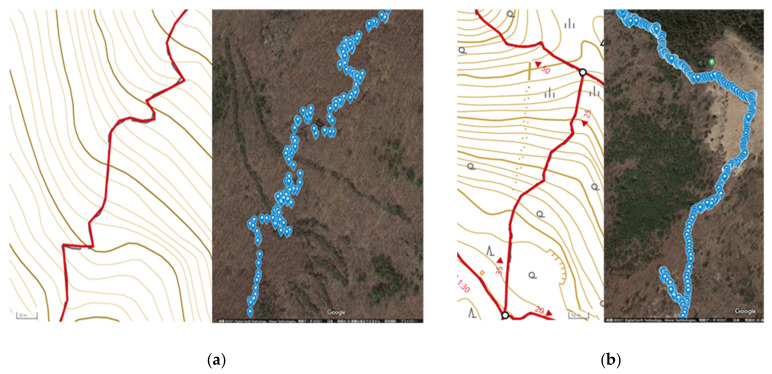
The comparison of log of the actual route of Mt. Kentoku. (**a**) The actual route and the GPS-recorded route in the mountainous area. (**b**) The actual route and the GPS-recorded route in the open and clear area.

Figure 10 shows the comparison between (a) the actual route and the GPS-recorded route in the mountainous area, and (b) the actual route and the GPS-recorded route in the open and clear areas.

**Figure 11:**
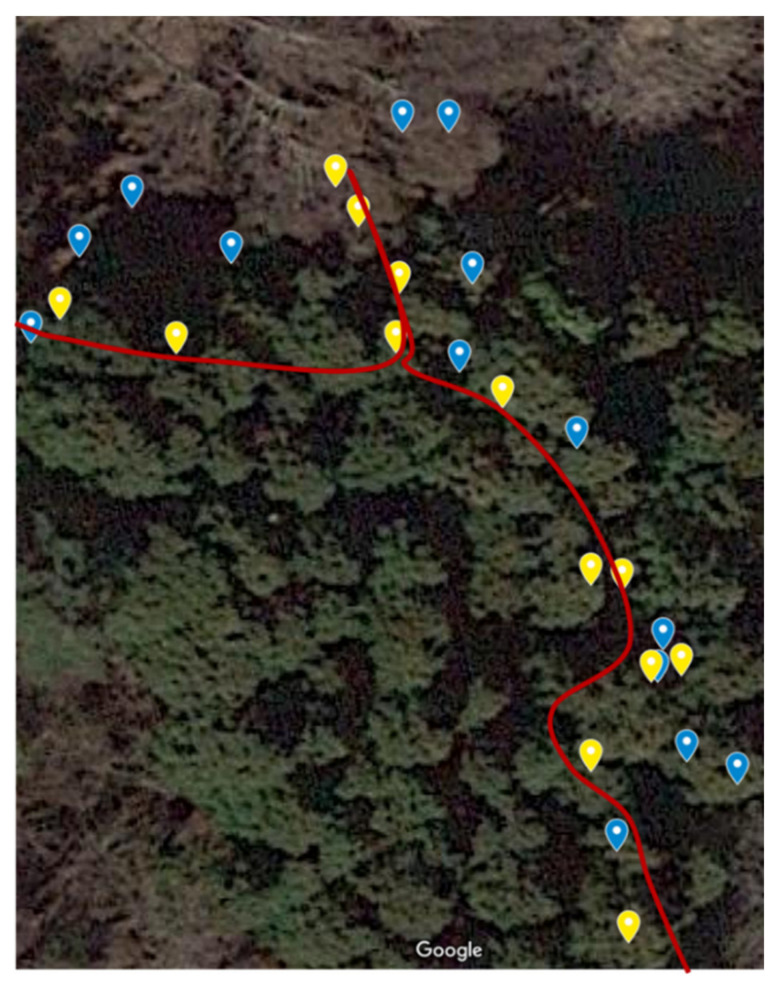
Estimated position in Mt. Kentoku (red line: actual route, blue dot: GPS record, yellow dot: estimated position).

From Figure 10, it can be seen that the GPS data follow the actual route relatively well throughout this experiment. However, the data in the mountainous areas are slightly scattered compared with the data in the open and clear areas. This because the mountain road is complicated until it reaches the ridge. However, the data are still not accurate when compared with the actual route. The estimated location information is shown in Figure 11. The numerical data of latitude/longitude and error distance are illustrated in Table 3.

Figure 11 displays the actual route (red line), the GPS record (blue points), and the position after the estimation process (yellow points). The yellow points are more in line with the actual route than the blue points. This is because the yellow point has linearity from the correlation of data before and after, compared with the blue point, which only records each GPS position as a jump value. However, the effect of the estimation process in this study was limited owing to good GPS reception throughout the entire activity.

## 5. Conclusions

In today’s world, where many mountaineers rely on the use of digital online devices, we emphasize the importance of offline device use. This study aimed to reduce the number of distress incidents in mountains by correctly estimating an individual’s location. There are not many other methods that correct GPS errors from the point of view of radio waves. Hence, we have presented a prospective software that calculates position information from a database. This study is expected to be a new location-correction technology.

In this method, data for all positions were used, as sufficient data are needed to derive the results. However, by adding to the method the process of removing obviously erroneous data [20], it is possible to obtain more accurate position estimates.

The method used in this study is designed to be used by pedestrians, because it is not generalizable to vehicles such as cars, trains, or airplanes, which can accelerate and suddenly stop. In the future, we aim to add more conditions to this method and expand the range of its effectiveness with new methods [21].

## Figures and Tables

**Table 1:** Spec of Raspberry Pi 3 Model B+.

CPU	ARM Cortex-A53 (1.4 GHz Quad-Core)
RAM	1 GB
DPC	2 W
OS	Debian 11.0 (bullseye)

**Table 2:** Latitude/longitude and error distance in Figure 7b.

Longitude	Latitude	Error (m)
35.62771	139.4649	2
35.62763	139.4648	5
35.62754	139.4648	6
35.62736	139.4649	0

**Table 3:** Latitude/longitude and error distance (blue: GPS record, yellow: estimated position).

Longitude	Latitude	Error (m)	Longitude	Latitude	Error (m)
35.80406	138.7152	1	35.80401	138.7152	2
35.8041	138.7152	6	35.8041	138.7152	1
35.80409	138.7153	7	35.80414	138.7152	4
35.80414	138.7152	2	35.80415	138.7152	3
35.80416	138.7152	2	35.80419	138.7152	0
35.80426	138.7152	1	35.80419	138.7152	1
35.8043	138.7151	4	35.80428	138.7151	0
35.80434	138.7151	6	35.80431	138.7151	0
35.80442	138.7151	4	35.80434	138.7151	1
35.80442	138.7151	8	35.80439	138.715	1
35.80435	138.715	9	35.80437	138.715	0
35.80438	138.7149	4	35.80431	138.7149	0
35.80436	138.7149	1	35.80433	138.7149	1

## Data Availability

Not applicable.
